# Postoperative blood pressure deficit and acute kidney injury progression in vasopressor-dependent cardiovascular surgery patients

**DOI:** 10.1186/s13054-016-1253-1

**Published:** 2016-03-24

**Authors:** Shinjiro Saito, Shigehiko Uchino, Masanori Takinami, Shoichi Uezono, Rinaldo Bellomo

**Affiliations:** Intensive Care Unit, Department of Anesthesiology, Jikei University School of Medicine, 3-19-18, Nishi-Shinbashi, Minato-ku, Tokyo 105-8471 Japan; Department of Intensive Care, Austin Hospital, 145 Studley Road, Melbourne, VIC 3084 Australia

**Keywords:** Blood pressure, Acute kidney injury, Blood pressure target, Relative hypotension, Perfusion pressure, Critical care

## Abstract

**Background:**

In vasopressor-dependent patients who had undergone cardiovascular surgery, we examined whether those with progression of acute kidney injury (AKI) had a greater difference (deficit) between premorbid and within-ICU hemodynamic pressure-related parameters compared to those without AKI progression.

**Methods:**

We assessed consecutive adults who underwent cardiovascular surgery and who stayed in our ICU for at least 48 hours and received vasopressor support for more than 4 hours. We obtained premorbid and vasopressor-associated, time-weighted average values for hemodynamic pressure-related parameters (systolic [SAP], diastolic [DAP], and mean arterial pressure [MAP]; central venous pressure [CVP], mean perfusion pressure [MPP], and diastolic perfusion pressure [DPP]) and calculated deficits in those values. We defined AKI progression as an increase of at least one Kidney Disease: Improving Global Outcomes stage.

**Results:**

We screened 159 patients who satisfied the inclusion criteria and identified 76 eligible patients. Thirty-six patients (47 %) had AKI progression. All achieved pressure-related values were similar between patients with or without AKI progression. However, deficits in DAP (*P* = 0.027), MPP (*P* = 0.023), and DPP (*P* = 0.002) were significantly greater in patients with AKI progression.

**Conclusions:**

Patients with AKI progression had greater DAP, MPP, and DPP deficits compared to patients without AKI progression. Such deficits might be modifiable risk factors for the prevention of AKI progression.

## Background

Blood pressure (BP) is an important determinant of renal perfusion. Therefore, achieving optimal BP in vasopressor-dependent patients is considered important for preventing the progression of acute kidney injury (AKI). Although international consensus guidelines recommend adjusting BP targets according to premorbid BP [1], there are limited studies investigating the putative benefits of adjusting BP targets according to premorbid levels in the clinical setting.

A recent multicenter randomized control trial showed no significant difference in mortality outcome in patients with septic shock undergoing resuscitation with a mean arterial pressure (MAP) target of either 80 mm Hg to 85 mm Hg (high-target group) or 65 mm Hg to 70 mm Hg (low-target group) [2]. However, that study also showed that patients with chronic hypertension in the high-target group had a lower incidence of AKI or requirement for renal replacement therapy. An observational cohort study also investigated mean deficits between premorbid and actual mean perfusion pressure (MPP) in vasopressor-dependent patients in the intensive care unit (ICU) (“relative hypotension”) and reported an association with subsequent AKI [3]. To our knowledge, no other studies have assessed the association of such relative hypotension and AKI progression in patients who have undergone cardiovascular surgery and are receiving vasopressor drugs [4]. In addition, a recent study reported an association of low diastolic BP and septic AKI [5], but no studies have investigated the association of relative *diastolic* hypotension and AKI progression.

Accordingly, we investigated differences between premorbid and achieved hemodynamic parameter levels (systolic arterial pressure [SAP], diastolic arterial pressure [DAP], MAP, MPP, and diastolic perfusion pressure [DPP]) in vasopressor-dependent patients to assess whether patients with AKI progression had greater pressure deficits compared to those without AKI progression. We specifically focused on patients who had undergone cardiovascular surgery and whose premorbid central venous pressure (CVP) could be estimated accurately according to preoperative echocardiography, to enable a more precise estimate of perfusion pressure.

## Methods

We conducted a retrospective observational study in a 20-bed ICU at a university hospital in Tokyo, Japan. The hospital ethics committee of Jikei University School of Medicine approved the study protocol, and the need for informed consent was waived because of the retrospective design of the study.

### Study patients

All patients who were admitted to our ICU between January 2010 and December 2013 were screened retrospectively. Consecutive patients who were 18 years of age or older, had had cardiovascular surgery, stayed in the ICU for at least 48 hours, and required vasopressor support for more than 4 hours were identified from our ICU database. We excluded patients who were on extracorporeal membrane oxygenation (ECMO) or intra-aortic balloon pump (IABP), were on long-term chronic dialysis, were readmitted to the ICU during the same hospitalization period, or required spinal perfusion pressure-guided therapy to prevent spinal cord ischemia [6].

### Premorbid baseline hemodynamic parameters

We collected three recent BP values measured in the outpatient clinic. If the number of BP values was insufficient (less than three), we used BP values noted on the ward charts before surgery for elective cases. BP values were excluded if there was any significant escalation of antihypertensive or diuretic therapy before BP measurements or any acute renal impairment during the period of BP observations.

Premorbid baseline BP was defined as the mean of the three recent values. MAP was estimated from SAP and DAP as DAP + (SAP–DAP)/3. Premorbid baseline CVP was estimated using inferior vena cava (IVC) parameters (diameter and collapse) derived from outpatient echocardiography examinations, according to the criteria recommended by the 2010 American Society of Echocardiography guidelines [7]: the IVC diameter 2.1 cm or less and collapse more than 50 % correlates with CVP (mean right atrial pressure) of 3 mmHg, the IVC diameter 2.1 cm or less with collapse 50 % or less or IVC diameter more than 2.1 cm with collapse more than 50 % correlates with CVP of 8 mmHg, and the IVC diameter more than 2.1 cm and collapse 50 % or less correlates with CVP of 15 mmHg. Premorbid baseline DPP and MPP were calculated from premorbid baseline DAP and MAP and estimated CVP values (DPP = DAP–CVP; MPP = MAP–CVP). We do not have a formal protocol for a target MAP for management of patients with shock, but we usually aim for a target MAP of 65 or 70 mmHg.

### Other study variables

We defined T0 as the time when vasopressor support was started. We collected SAP, DAP, MAP, and CVP values every 15 minutes, extracted from the patient information system (PIMS®, Philips Respironics, Tokyo, Japan). MPP and DPP were calculated for each data point. These data were collected from T0 until 72 hours later or until the time vasopressor support was stopped. We derived time-weighted average (TWA) values during vasopressor support from collected data for each patient. TWA was calculated as follows:

$$ \mathrm{T}\mathrm{W}\mathrm{A} = \left({\mathrm{t}}_1{\mathrm{X}}_1 + {\mathrm{t}}_2{\mathrm{X}}_2+\dots + {\mathrm{t}}_{\mathrm{n}}{\mathrm{X}}_{\mathrm{n}}\right)/\left({\mathrm{t}}_1 + {\mathrm{t}}_2+\dots + {\mathrm{t}}_{\mathrm{n}}\right) $$

where X_n_ is the value of the variable of interest during the n^th^ interval, and t_n_ is the duration of the n^th^ interval. The percent deficit in TWA parameters in relation to baseline parameters was determined as % parameter deficit. For example, we calculated %MPP deficit as (achieved MPP– baseline MPP)/baseline MPP.

We used the Kidney Disease: Improving Global Outcomes (KDIGO) creatinine and urine output criteria for staging and definition of AKI [8]. Changes in serum creatinine were compared to the premorbid creatinine level, which was defined as the latest value before operation. KDIGO stage changes from T0 were assessed during the initial 72 hours. We defined an increase of at least one KDIGO stage from T0 as progression of AKI (AKI+) and no KDIGO stage change as no progression of AKI (AKI–). TWA vasopressor rates were derived from noradrenaline infusion rates because our ICU uses noradrenaline almost exclusively for vasopressor therapy. In rare cases of profound shock refractory to high-dose noradrenaline, vasopressin was used.

We collected demographic data (age, sex, body weight, baseline creatinine level), along with data regarding pre-ICU use of antihypertensive drugs, mechanical ventilation, Acute Physiology and Chronic Health Evaluation (APACHE) II score [9], Sepsis-related Organ Failure Assessment (SOFA) score [10] on the day when vasopressor support was started, serum lactate level at T0, mixed venous oxygen saturation (SvO_2_) and cardiac index at T0, admission type, surgery type, intraoperative information (duration of operation, blood loss, fluid balance), use of vasopressin, exposure of nephrotoxic drugs, and ICU and hospital lengths of stay. Surgery type was categorized as cardiac surgery or vascular surgery. Coronary artery bypass graft and surgery using cardiopulmonary bypass (valve repair, thoracic aorta aneurysm, or other) was categorized as cardiac surgery. Aortic surgery without cardiopulmonary bypass, which included both open repair and endovascular repair, was categorized as vascular surgery.

### Statistical analysis

We compared hemodynamic parameters and other variables between the AKI+ group and the AKI– group. Quantitative parameters were reported as median and interquartile range (25^th^ to 75^th^ percentile) and were compared using the Mann-Whitney *U* test. Qualitative parameters were expressed as number and percentage and were compared using the chi-square test or Fisher exact test, as appropriate. A *P* value of less than 0.05 was considered statistically significant. A commercially available statistical package (SPSS 19.0, IBM Corp., Armonk, NY, USA) was used for all statistical analyses.

## Results

**Fig. 1:**
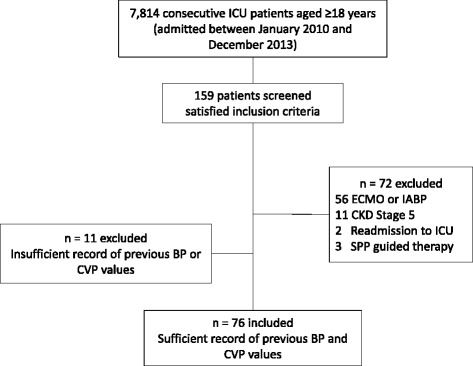
Flow chart of study patients. *BP* blood pressure, *CKD* chronic kidney disease, *CVP* central venous pressure, *ECMO* extracorporeal membrane oxygenation, *IABP* intra-aortic balloon pump, *ICU* intensive care unit, *SPP* spinal perfusion pressure

A total of 7,814 consecutive adult patients were admitted to our ICU between January 2010 and December 2013. We screened 159 patients who satisfied the inclusion criteria and identified 76 eligible patients (Fig. 1). Table 1 lists demographic and clinical characteristics of these patients. Thirty-six patients (47 %) had an increase of at least one KDIGO stage from T0 to 72 hours (AKI+ group). Baseline creatinine level was higher in the AKI+ group (0.88 mg/dL vs. 1.07 mg/dL; *P* = 0.018). Surgery types were similar between the AKI+ and AKI– groups (*P* = 0.373). Fifty-one patients (all cardiac surgery) had a pulmonary artery catheter. Cardiac index (2.6 L/min/m^2^vs. 2.7 L/min/m^2^, *P* = 0.593) and SvO_2_ (67 % vs. 72 %, *P* = 0.269) were similar between the two groups.

**Table 1:** Patient characteristics

	All patients	No KDIGO stage shift (AKI–)	≥1 KDIGO stage shift (AKI+)	*P* value
Number of patients	76	40	36	
Age, years	71 (63-77)	69 (62-75)	74 (66-78)	0.057
Male sex	53 (70)	28 (70)	25 (69)	0.958
Weight, kg	61 (52-70)	64 (54-70)	59 (51-67)	0.318
Baseline creatinine, mg/dL	0.92 (0.76-1.13)	0.88 (0.72-1.05)	1.07 (0.84-1.39)	0.018
Antihypertensive drugs	61 (80)	31 (78)	30 (83)	0.523
Emergency surgery	4 (5)	1 (3)	3 (8)	0.255
Cardiac surgery	52 (68)	31 (78)	21 (58)	0.730
Surgery type				0.373
*Cardiac surgery*				
CABG	14 (18)	10 (25)	4 (11)	
Valve replacement	21 (28)	13 (33)	8 (22)	
TAA	8 (11)	3 (8)	5 (14)	
Mixed	7 (9)	4 (10)	3 (8)	
Other	2 (3)	1 (3)	1 (3)	
*Vascular surgery*				
Open aortic repair	16 (21)	5 (13)	11 (31)	
Endovascular aortic repair	8 (11)	4 (10)	4 (11)	
Duration of operation, hours	7.08 (6.00-8.68)	7.03 (5.66-8.91)	7.08 (6.00-8.65)	0.992
Intraoperative blood loss, mL	405 (203-841)	373 (143-675)	505 (263-949)	0.080
Intraoperative fluid balance, mL	2750 (2150-5260)	2675 (2146-4650)	2925 (2150-5595)	0.655
Mechanical ventilation	68 (90)	35 (88)	33 (92)	0.555
APACHE II score	16 (12-20)	15 (11-18)	17 (14-21)	0.046
SOFA score total	8 (6-9)	7 (5-9)	8 (7-9)	0.290
Cardiovascular	3 (3-4)	3 (3-4)	3 (3-4)	0.847
Respiratory	1 (1-2)	1 (1-2)	1.5 (1-2)	0.818
Liver	0 (0-1)	0 (0-1)	0 (0-1)	0.554
Renal	0 (0-1)	0 (0-1)	1 (0-1)	0.005
Coagulation	2 (1-2)	2 (1-2)	2 (1-2)	0.527
Central nerves	0 (0-0)	0 (0-0)	0 (0-0)	0.053
Lactate, mmol/L	1.7 (1.1-2.4)	1.6 (1.0-2.2)	1.8 (1.1-2.7)	0.250
Exposed to nephrotoxins in ICU	2 (3)	1 (3)	1 (3)	0.940
ICU length of stay, days	3.8 (2.8-5.8)	3.7 (2.8-5.5)	4.2 (2.8-5.8)	0.240
Hospital length of stay, days	30 (23-39)	31 (23-37)	29 (21-44)	0.750
Hospital mortality	3 (4)	2 (5)	1 (3)	0.619

Quantitative parameters are reported as median (interquartile range; 25^th^–75^th^ percentile). Qualitative parameters are expressed as number (%)

*AKI* acute kidney injury, *APACHE II* Acute Physiology and Chronic Health Evaluation II, *CABG* coronary artery bypass graft, *ICU* intensive care unit, *KDIGO* Kidney Disease: Improving Global Outcomes, *SOFA* Sequential Organ Failure Assessment, *TAA* thoracic aorta aneurysm

Table 2 lists comparisons of hemodynamic baseline values and values achieved during vasopressor support between the AKI+ and AKI– groups. Data on premorbid BP were from measurements made in the outpatient clinic (83 % of BP values) and in the ward before elective operation (17 % of BP values). Overall, the median time on vasopressor therapy was 1.3 days, and the TWA noradrenaline rate was 0.08 μg/kg/minute. Patients in the AKI+ group had higher baseline MPP (91 mm Hg vs. 85 mm Hg; *P* = 0.035), baseline DPP (73 mmHg vs. 67 mmHg; *P* = 0.047) and lower baseline CVP (*P* = 0.018). Both groups achieved similar TWA parameters for all hemodynamic pressures.

**Table 2:** Baseline and achieved hemodynamic parameters and variables during vasopressor support

	All patients	No KDIGO stage shift (AKI–)	≥1 KDIGO stage shift (AKI+)	*P* value
Baseline SAP, mm Hg	130 (120-138)	130 (117-137)	131 (120-142)	0.592
Baseline DAP, mm Hg	74 (67-84)	73 (62-82)	79 (69-85)	0.163
Baseline MAP, mm Hg	92 (86-101)	91 (84-99)	97 (87-103)	0.154
Baseline CVP, mm Hg	3 (3-8)	3 (3-8)	3 (3-8)	0.018
3 mm Hg	48 (63)	21 (53)	27 (75)	
8 mm Hg	21 (28)	12 (30)	9 (25)	
15 mm Hg	7 (9)	7 (17)	0 (0)	
Baseline MPP, mm Hg	89 (82-97)	85 (76-93)	91 (84-99)	0.035
Baseline DPP, mm Hg	70 (63-81)	67 (55-76)	73 (64-81)	0.047
Time on vasopressor, days	1.3 (0.7-2.7)	1.2 (0.7-2.3)	1.4 (0.7-2.8)	0.303
TWA vasopressor rate, μg/kg/min	0.08 (0.04-0.12)	0.08 (0.05-0.12)	0.08 (0.04-0.12)	0.685
Vasopressin	5 (7)	2 (5)	3 (8)	0.558
TWA-SAP achieved, mm Hg	118 (110-128)	115 (107-128)	120 (114-129)	0.170
TWA-DAP achieved, mm Hg	58 (53-62)	58 (54-62)	57 (53-62)	0.803
TWA-MAP achieved, mm Hg	74 (70-78)	73 (70-78)	75 (72-79)	0.323
TWA-CVP achieved, mm Hg	8.0 (5.7-9.6)	7.7 (5.6-9.4)	8.6 (5.8-9.7)	0.344
TWA-MPP achieved, mm Hg	66 (62-70)	65 (61-70)	67 (63-71)	0.533
TWA-DPP achieved, mm Hg	50 (46-53)	50 (47-54)	50 (46-54)	0.892

Quantitative parameters are reported as median (interquartile range; 25^th^–75^th^ percentile). Qualitative parameters are expressed as number (%)

*AKI* acute kidney injury, *CVP* central venous pressure, *DAP* diastolic arterial pressure, *DPP* diastolic perfusion pressure, *KDIGO* Kidney Disease: Improving Global Outcomes, *MAP* mean arterial pressure, *MPP* mean perfusion pressure, *SAP* systolic arterial pressure, *TWA* time-weighted average

Table 3 lists the AKI stage at T0, maximum AKI stage and progression during the initial 72 hours and use of continuous renal replacement therapy (CRRT) during ICU stay. The AKI stage at T0 was similar between the AKI+ and AKI– groups (*P* = 0.408). The number of patients who had maximum AKI progression was 18 (50 %) on day 1, 14 (39 %) on day 2, and four (11 %) on day 3.

**Table 3:** Staging of AKI

	All patients	No KDIGO stage shift (AKI–)	≥1 KDIGO stage shift (AKI+)	*P* value
AKI stage at T0				0.408
No AKI	66 (87)	33 (83)	33 (92)	
Stage 1	5 (7)	4 (10)	1 (3)	
Stage 2	5 (7)	3 (8)	2 (6)	
Maximum AKI stage				<0.001
No AKI	33 (43)	33 (83)	0 (0)	
Stage 1	24 (32)	4 (10)	20 (56)	
Stage 2	11 (15)	3 (8)	8 (22)	
Stage 3	8 (11)	0 (0)	8 (22)	
AKI stage progression				<0.001
No change	40 (53)	40 (100)	0 (0)	
One stage	23 (30)	0 (0)	23 (64)	
Two stages	7 (9)	0 (0)	7 (19)	
Three stages	6 (8)	0 (0)	6 (17)	
CRRT during ICU stay	5 (7)	1 (3)	4 (11)	0.131

Qualitative parameters are expressed as number (%)

*AKI* acute kidney injury, *CRRT* continuous renal replacement therapy, *ICU* intensive care unit, *KDIGO* Kidney Disease: Improving Global Outcomes, *T0* the time when vasopressor support was started

**Fig. 2:**
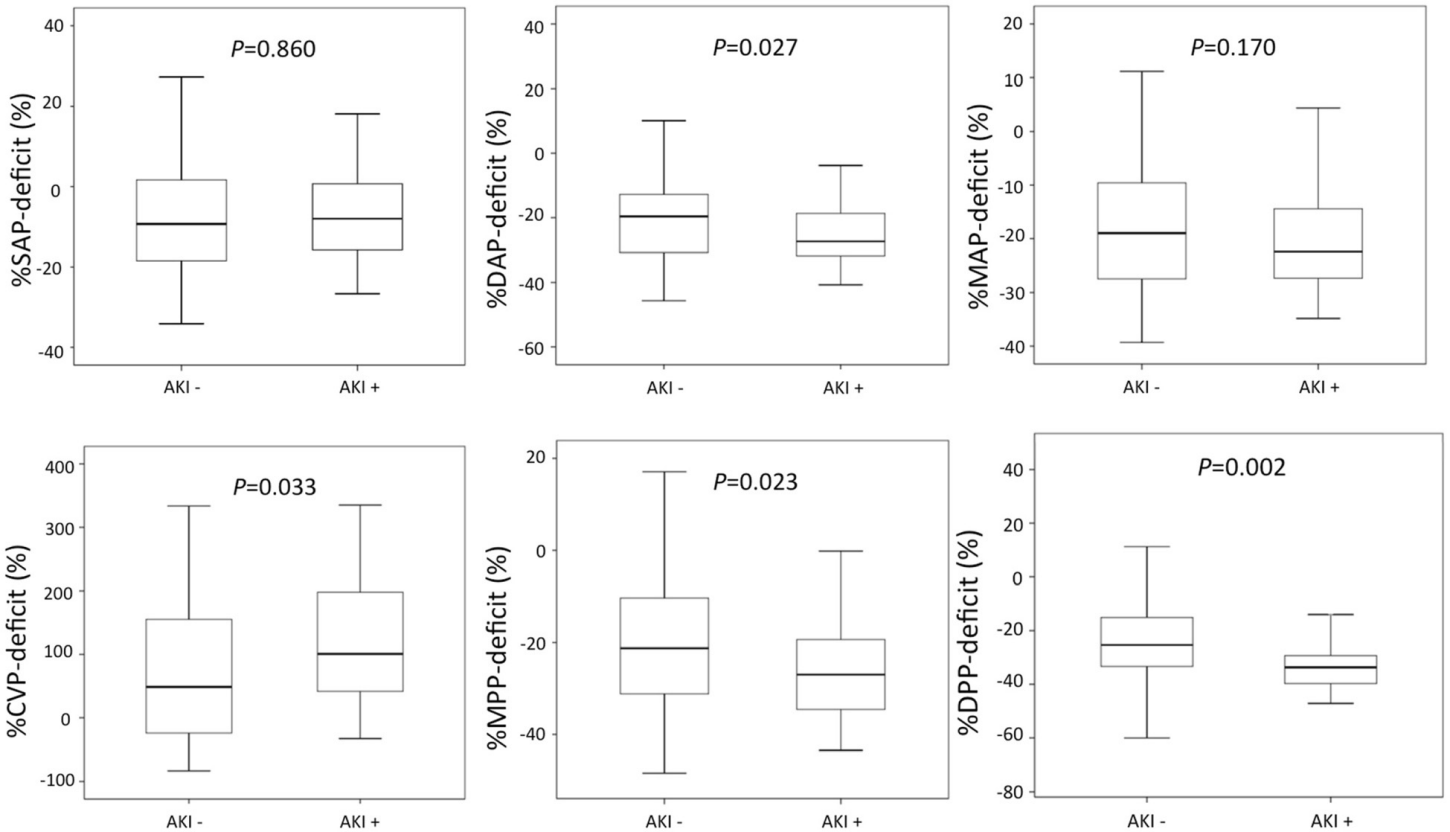
Comparison of % parameter deficits between patients with progression of AKI (AKI+) and without progression of AKI (AKI–). *AKI* acute kidney injury, *AKI+* increase of at least one KDIGO stage shift, *AKI–* no KDIGO stage shift, *CVP* central venous pressure, *DAP* diastolic arterial pressure, *DPP* diastolic perfusion pressure, *KDIGO* Kidney Disease: Improving Global Outcomes, *MAP* mean arterial pressure, *MPP* mean perfusion pressure, *SAP* systolic arterial pressure

**Fig. 3:**
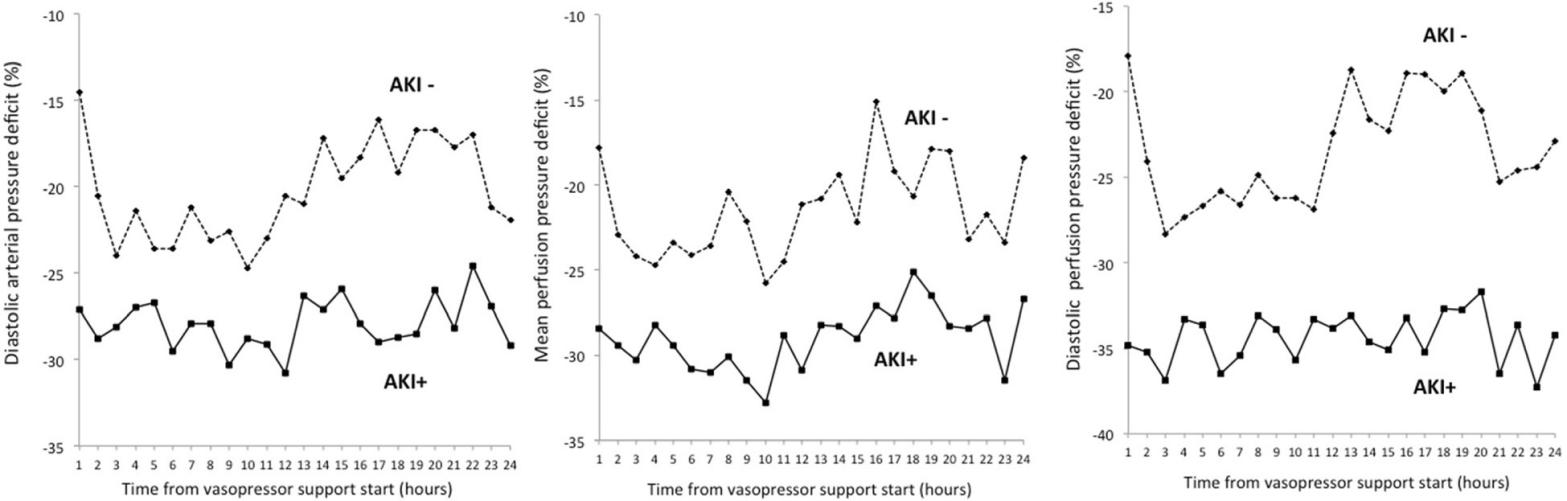
Hemodynamic deficit change during the first 24 hours of vasopressor support. Diastolic arterial pressure (DAP) deficit, mean perfusion pressure (MPP) deficit, and diastolic perfusion pressure (DPP) deficit for AKI+ (*solid lines*) and AKI– (*dashed lines*), respectively. Data are median values. *AKI* acute kidney injury, *AKI+* increase of at least one KDIGO stage shift, *AKI–* no KDIGO stage shift, *KDIGO* Kidney Disease: Improving Global Outcomes

Figure 2 shows % parameter deficits between the AKI+ and AKI– groups, demonstrating that %MAP deficit and %SAP deficit did not differ between the two groups. In contrast, %DAP deficit (*P* = 0.027), %MPP deficit (*P* = 0.023) and %DPP deficit (*P* = 0.002) were significantly greater in the AKI+ group compared to the AKI– group. Figure 3 shows hourly changes in %DAP, %MPP, and %DPP deficits in the AKI+ and AKI– groups. The AKI+ group had consistently greater deficits of all three perfusion pressures in the first 24 hours of vasopressor support compared with the AKI– group. Overall, 21.7 % and 23.8 % of the %MPP and %DPP deficits, respectively, were due to an increase in CVP, with no difference between the AKI+ and AKI– groups.

## Discussion

### Key findings

We investigated whether, in patients who had undergone cardiovascular surgery and were receiving vasopressor support, the time-weighted difference between premorbid and in-ICU pressure-related parameters (the degree of relative hypotension) was greater among those who developed AKI progression compared with those who did not. We observed that although patients with AKI progression had equivalent MAP, DAP, and MPP to those who did not, they had significantly greater DAP, MPP, and DPP deficits.

### Comparison to previous studies

Selection of an effective BP target to manage vasopressor-dependent patients has been a controversial issue for decades. With respect to septic shock, a large randomized clinical trial (N = 776) compared protocols targeting MAPs of either 65 mm Hg to 70 mm Hg or 80 mm Hg to 85 mm Hg for 5 days and found no significant difference in mortality [2]. However, chronic hypertension was reported in 44 % of patients, and in a predefined subgroup analysis of these patients, targeting a higher MAP was associated with a lower rate of serum creatinine doubling and renal replacement therapy requirement. A recent review of data related to MAP targets in septic shock identified seven comparative studies and suggested that a MAP target of 65 mm Hg is usually sufficient in patients with septic shock, but a MAP of approximately 75 mm Hg to 85 mm Hg may prevent AKI development in patients with chronic hypertension [11].

Fewer studies exist for types of shock other than septic shock. In a case-control study in noncritically ill ward patients, those who developed AKI were more likely to have had relative hypotension than control patients [12]. In an observational study of patients during cardiopulmonary bypass in cardiac surgery, the duration and degree to which MAP was below the cerebral autoregulation threshold was independently associated with AKI, but absolute MAP was not [13]. To the best of our knowledge, there are only two previous studies that evaluated the impact of changes from the premorbid BP level on subsequent AKI development or progression in critically ill patients with shock [3, 14]. One observational study comprised 51 consecutive heterogeneous patients requiring vasopressors and compared premorbid MPP estimated from premorbid BP and CVP with MPP during resuscitation [3]. The incidence of AKI progression was greater among patients with greater MPP deficits. The other observational study comprised 107 patients with septic shock and investigated the association of change from premorbid values with AKI progression [14]. Median MAP deficit was similar for patients with or without severe AKI; however, median MPP deficit was greater in patients with severe AKI. These results are consistent with those found in the present study.

We observed a greater DAP deficit (*P* = 0.027) and DPP deficit (*P* = 0.002) in the AKI+ group. We also observed a lower CVP deficit in the AKI+ group (*P* = 0.033). A recent retrospective study of 137 septic patients in the ICU showed associations between lower DAP or higher CVP and AKI but not between MAP or cardiac output and AKI [5]. The present study is the first to report an association between changes from premorbid DAP and AKI progression. In the present study, 23.8 % of the %DPP deficit was due to an increase in CVP, and 76.2 % was due to a decrease in DAP. These results suggest that a role of both DAP and back pressure (renal venous pressure) are potentially important in the development of AKI.

### Study implications

Previous studies have reported an association between greater premorbid MAP and a shift of the kidney’s autoregulatory range to the right [15]. Our present results imply that the autoregulation of renal blood flow may be more strongly affected by MPP and DPP than MAP because premorbid MPP and DPP were higher in AKI+ group but premorbid MAP was similar between groups. Particularly, deficit in diastolic perfusion may be the major factor responsible for decreased renal perfusion and function. Because renal vascular resistance is low, as evidenced by a positive diastolic blood flow velocity [16, 17], DPP might be a key determinant of renal perfusion. This raises the possibility that increasing diastolic blood flow and decreasing back-pressure determinants (CVP or renal venous pressure) may be important future targets for hemodynamic manipulation in these patients. This study could not assess a causal relationship between hemodynamic parameter deficits and AKI progression. Further studies are needed to clarify whether it is possible to prevent subsequent AKI progression with hemodynamic interventions, and what would be the targets of DAP, MPP and DPP deficits.

### Study strengths and limitations

This is the first study to investigate changes from premorbid to in-ICU levels of key pressures in patients with shock after cardiovascular surgery and the first to identify which hemodynamic parameters differ in patients who develop AKI progression. We focused on patients who had undergone cardiovascular surgery for whom we could estimate premorbid CVP accurately according to preoperative echocardiography. In addition, this is the first study to demonstrate the importance not only of MPP deficit but also of DPP deficit as possible risk factors for subsequent deterioration of renal function.

There are several limitations in the present study. First, this was single center in design, with a small sample size. We excluded patients who stayed in the ICU for shorter than 48 hours, since our study design required a long observational period, enough to diagnose AKI progression. We also excluded patients who received mechanical cardiovascular support (ECMO or IABP), to avoid the impact of mechanical support on renal perfusion. However, we believe that such exclusion made our study patients more homogenous to study an association between systemic hemodynamics and AKI progression in patients receiving vasopressors. However, the incidence of AKI and outcomes in patients in our ICU are consistent with those of studies reporting the epidemiology of AKI in patients undergoing cardiovascular surgery [18, 19]. The sample size of our study was larger than that of the only previous study of patients with nonspecific shock [3]. In addition, the median achieved MAP of 74 mm Hg in our study patients was quite similar to that reported among vasopressor-dependent septic shock patients in the previous major randomized trial [20] and in a recent observational study of the association between systematic hemodynamics and septic AKI [5]. The median achieved CVP of 8 mm Hg was also the same as the lower limit level recommended in the current guidelines for septic shock [21] and slightly lower than that reported in a recent randomized trial of early goal-directed therapy [22].

It is unclear whether the methods of BP and CVP estimation used in the present study represent true premorbid BP and CVP values. However, we estimated baseline CVP by using inferior vena cava parameters according to the American Society of Echocardiography guidelines [7]. This approach might make the premorbid CVP value more accurate than that reported in previous studies [3, 14]. Finally, we studied only a cohort of patients with shock after cardiovascular surgery. Therefore, our findings may not apply to patients with shock due to other types of critical illness.

## Conclusions

We found that despite equivalent unadjusted MAP, DAP, and MPP values, vasopressor-dependent patients who had undergone cardiovascular surgery with AKI progression had greater DAP, MPP, and DPP deficits compared to patients without AKI progression. These results suggest that deficits of DAP, MPP, and DPP might be considered as adjustable target hemodynamic parameters to prevent subsequent AKI progression. In addition, our findings suggest the need to further investigate the relation between diastolic perfusion and AKI progression in patients with circulatory shock.

## Key messages

- Deficits of DAP, MPP, and DPP might be considered as adjustable target hemodynamic parameters to prevent subsequent AKI progression.
- There is the need to further investigate the relation between diastolic perfusion and AKI progression in patients with circulatory shock.

## Abbreviations

AKI: acute kidney injury
APACHE II: Acute Physiology and Chronic Health Evaluation II
BP: blood pressure
CRRT: continuous renal replacement therapy
CVP: central venous pressure
DAP: diastolic arterial pressure
DPP: diastolic perfusion pressure
ECMO: extracorporeal membrane oxygenation
IABP: intra-aortic balloon pump
ICU: intensive care unit
IVC: inferior vena cava
KDIGO: Kidney Disease: Improving Global Outcomes
MAP: mean arterial pressure
MPP: mean perfusion pressure
SAP: systolic arterial pressure
SOFA: Sepsis-related Organ Failure Assessment
SvO_2_: mixed venous oxygen saturation
TWA: time-weighted average
